# *In Vitro* Murine Hematopoiesis Supported by Signaling from a Splenic Stromal Cell Line

**DOI:** 10.1155/2018/9896142

**Published:** 2018-12-25

**Authors:** Hong Kiat Lim, Pravin Periasamy, Helen C. O'Neill

**Affiliations:** ^1^Clem Jones Research Centre for Regenerative Medicine, Bond University, Gold Coast, Australia; ^2^Department of Microbiology, Yong Loo Lin School of Medicine, National University of Singapore, Singapore

## Abstract

There are very few model systems which demonstrate hematopoiesis *in vitro*. Previously, we described unique splenic stromal cell lines which support the *in vitro* development of hematopoietic cells and particularly myeloid cells. Here, the 5G3 spleen stromal cell line has been investigated for capacity to support the differentiation of hematopoietic cells from progenitors *in vitro*. Initially, 5G3 was shown to express markers of mesenchymal but not endothelial or hematopoietic cells and to resemble perivascular reticular cells in the bone marrow through gene expression. In particular, 5G3 resembles CXCL12-abundant reticular cells or perivascular reticular cells, which are important niche elements for hematopoiesis in the bone marrow. To analyse the hematopoietic support function of 5G3, specific signaling pathway inhibitors were tested for the ability to regulate cell production *in vitro* in cocultures of stroma overlaid with bone marrow-derived hematopoietic stem/progenitor cells. These studies identified an important role for Wnt and Notch pathways as well as tyrosine kinase receptors like c-KIT and PDGFR. Cell production in stromal cocultures constitutes hematopoiesis, since signaling pathways provided by splenic stroma reflect those which support hematopoiesis in the bone marrow.

## 1. Introduction

Multiple interactions occur between HSC and the stromal niche in the bone marrow, acting as essential triggers for hematopoiesis. Known signaling events include CXCL12/CXCR4 and SCF/c-KIT receptor-ligand interactions and Wnt and Notch signaling [1–4]. It is well known that the soluble factors CXCL12 and SCF produced by perivascular reticular cells are important mediators of hematopoietic stem cell (HSC) migration and maintenance, respectively [1, 5]. Wnt signaling is crucial for HSC self-renewal, and HSC from *Wnt3a^−/−^* mice are severely compromised in repopulation capacity [6]. The role of Notch in hematopoiesis *in vivo* is disputable. However, inhibition of Notch in cocultures of CD146^+^ perivascular cells with human HSC gave increased B cell development with fewer HSC, suggesting a role for Notch in HSC maintenance [2]. Despite a wealth of information on the signaling pathways and niches which support hematopoiesis in the bone marrow, there are very few examples whereby hematopoiesis can be induced *in vitro* either through provision of growth factors or through coculture with stromal niche cells.

This laboratory previously reported a stromal cell line 5G3 isolated from murine spleen which supports *in vitro* production of specific myeloid cell subsets [7]. This finding in relation to stromal cells in spleen evokes interest in the spleen as a hematopoietic niche, and of spleen as a secondary site supporting hematopoiesis. 5G3 stroma was shown to support transient production of myeloid precursors and long-term production of a novel distinct dendritic-like cell type, namely, “L-DC” [7]. Previous studies identified L-DC as highly endocytic with the ability to activate CD8^+^ T cells, but not CD4^+^ T cells [8, 9]. 5G3 provides contact-dependent support for hematopoiesis [8]. It is a clonal isolate of the long-term spleen culture STX3 and does not resemble mature endothelial or fibroblastic cells in gene expression, although it was shown to have weak ability compared with endothelial cells to form tube-like structures in Matrigel [10, 11]. This property has since been associated with pericytes and perivascular stromal cells [12].

The 5G3 stromal line has therefore been investigated further for marker expression to determine lineage origin in relation to mesenchymal and perivascular reticular cells as components of HSC niches described in the bone marrow. In order to address the question whether cocultures involving 5G3 support hematopoiesis in terms of differentiation of HSC to give progeny cells, as opposed to proliferation or expansion of hematopoietic cells, signaling pathways which regulate hematopoiesis in the bone marrow have been investigated. Inhibitors of known signaling pathways including Notch, Wnt, and the tyrosine kinases c-KIT and PDGFR have been tested in coculture assays for impact on cell production, maintenance of progenitors, and production of myeloid cells including L-DC.

## 2. Materials and Methods

### 2.1. Animals

Specific pathogen-free C57BL/6J (*H-2K^b^: CD45.2*) mice aged 4 to 8 weeks were obtained from the John Curtin School of Medical Research (JCSMR: Canberra, ACT, Australia). Animal experimentation was performed in accordance with the Australian Code for the Care and Use of Animals for Scientific Purposes (8th Edition, 2013). All procedures were approved by the Animal Experimentation Ethics Committee at the Australian National University (ANU: Canberra, ACT, Australia) under protocol A2013/11.

### 2.2. Stromal Cell Cultures

Spleen long-term cultures (LTC) have been established over many years in this laboratory through continuous culture of whole spleen cell suspension [13, 14]. These have been shown to comprise a stromal cell monolayer which continuously supports the production of distinct myeloid cell types. The STX3 stromal line was derived from a spleen LTC which ceased the production of hematopoietic cells over time and passage *in vitro* [10]. STX3 was cloned through single cell deposition using flow cytometry and then clones grown to confluence, recloned, and tested for hematopoietic support capacity. Several cloned lines including 5G3 studied here were selected as the hematopoietic supporter cell line [15]. 5G3 was originally classified as an early endothelial-like cell line on the basis of ability to form tube-like structures in Matrigel [16]. It did not, however, express markers of mature endothelial cells, and its lineage origin has been unclear.

Frozen stocks of 5G3 were banked so that experimentation has always involved cells passaged 3–4 times from frozen stocks. 5G3 cells grown from frozen stocks were cultured under conditions described previously and passaged by scraping cells [7]. Briefly, cells were cultured at 37°C in 5% CO_2_ in air with 95% humidity in Dulbecco's modified Eagle's medium (DMEM) (Sigma-Aldrich: Castle Hill, NSW, Australia) supplemented with 10% fetal calf serum (FCS), 5 × 10^−4^M 2-mercaptoethanol, 10 mM HEPES, 100 U/ml penicillin, 100ug/ml streptomycin, 4 mg/l glucose, 6 mg/l folic acid, 36 mg/l L-asparagine, and 116 mg/l L-asparagine hydrochloric acid (sDMEM). Trypsin treatment was used to dissociate stromal cells for experimentation.

### 2.3. Flow Cytometry

Cells were stained with antibodies diluted in fluorescence-activated cell sorting (FACS) buffer (DMEM/0.1% sodium azide/1% FCS). Antibody specific for Fc*γ*II/IIIR (CD32/CD16) (eBiosciences: San Diego, CA, USA) was used at 5 *μ*g/10^6^ cells in 1 ml to block nonspecific antibody binding to cells. Fluorochrome or biotin-conjugated antibodies for CD11b, CD11c, MHC-II, F4/80, CD3, B220, CD150, CD48, Ly6G, CD45.2, CD29, CD51, CD54, CD31, gp38, CD105, Thy1.2, Sca-1, VCAM1, CD140a, Flt3, NK1.1, CD19, Gr-1, Ter119, and c-Kit, as well as streptavidin-PE-Cy7, streptavidin-PE, and streptavidin-FITC, were purchased from BioLegend (San Diego, CA, USA). Staining with 1 *μ*g/ml propidium iodide (PI) (Sigma-Aldrich) was used to discriminate live cells. Isotype-matched control antibodies were used to set gates to assess specific antibody binding. Median fluorescence intensity (MFI) was calculated as the net change in median channel fluorescence for specific antibody above isotype control. Fluorescence minus one (FMO) controls were used to set gates for specific antibody binding in multicolour staining experiments. Flow cytometric analysis was carried out using BD FACSDiva (Becton Dickinson) and FlowJo software (Tree Star: Ashland, OR, USA).

### 2.4. Preparation of Bone Marrow Progenitors

Single cell suspensions were prepared by passing the bone marrow through a fine mesh sieve. Red blood cell lysis and lineage (Lin) depletion was carried out using MACS® magnetic bead technology (Miltenyi Biotec: Gladbach, Germany) as described previously [8, 17].

### 2.5. Isolation of Hematopoietic Stem and Progenitor Cells

Lineage-depleted (Lin^−^) bone marrow progenitors were prepared and stained with fluorochrome-conjugated antibodies to CD150, Flt3, c-Kit, and Sca-1 to delineate long-term- (LT-) HSC and multipotential progenitors (MPP) for sorting. Antibodies specific for lineage markers were used to gate out mature hematopoietic cells. LT-HSC were sorted as the CD150^+^Flt3^−^ subset of Lin^−^Sca-1^+^c-Kit^+^ (LSK) cells and MPP as the CD150^−^Flt3^+^ subset of LSK cells [17]. Sorting was performed on a BD FACSAria™ II cell sorter (Becton Dickinson).

### 2.6. Stromal Coculture Assays

To assess *in vitro* hematopoiesis, Lin^−^ bone marrow cells were overlaid at 1–5 × 10^4^ cells/ml above 5G3 monolayers of 80–90% confluency. LT-HSC and MPP were overlaid at 1–5 × 10^3^ cells/ml. Control cultures included stroma or Lin^−^ bone marrow cells only and LT-HSC or MPP cultured alone. In these controls, no hematopoietic cell survival was evident within 7 days of culture. Inhibitors used included Imatinib (inhibitor of FLT3, PDGFRA/B, and c-KIT tyrosine kinases; Sapphire Bioscience: Waterloo, NSW, Australia), Plerixafor (CXCR4 inhibitor/CXCL12 signaling; Sapphire Bioscience), XAV939 (WNT/*β*-catenin inhibitor; Sigma-Aldrich), WntC59 (PORCN inhibitor of WNT activation; Sigma-Aldrich), and DAPT (NOTCH/*γ*-secretase inhibitor; Sigma-Aldrich). These were titrated for concentration-dependent effects in trial experiments. They were replenished in cocultures at biweekly medium change. To assess cell production in cocultures, nonadherent cells were collected every 7 days by aspiration and replacement of medium.

### 2.7. Microscopy

Cell morphology was observed and photographed using an EVOS® FL digital fluorescence microscope (Electron Microscope Sciences: Hatfield, PA, USA) equipped with a Sony® ICX445 CCD camera (Sony: Minato, TKY, JP).

### 2.8. Transcriptome Analysis

Total RNA was prepared using an RNeasy mini kit (Qiagen: Clifton Hill, VIC, Australia) for transcriptome analysis using Murine Genome 430 v2 genechips (Affymetrix, Santa Clara, CA, USA). Double-stranded cDNA from extracted RNA was synthesised in a two-step process. First-strand cDNA was produced using T7-(dT)_24_ primers and Superscript II reverse transcriptase (Invitrogen Life Technologies: Mount Waverley, VIC, Australia). Second-strand cDNA was synthesised from the first. cRNA was then transcribed *in vitro* and biotin labelled from double-stranded DNA using the BioArray High Yield RNA Transcript Labelling Kit (Affymetrix). cRNA was hybridized to genechips which were washed and stained on the Affymetrix Fluidics station. The Affymetrix GeneArray® Scanner was used to analyse genechips.

### 2.9. Statistical Analysis

Data are presented as the mean ± standard error (SE) for sample size *n*. Statistical analysis involved a pairwise comparison of replicated cultures with controls. These were established at the same time and, in some cases, assayed at several time points. The statistical procedure therefore involved a Bonferroni correction to the significance level of Student's *t*-test (*p* ≤ 0.05), reflecting the fact that multiple comparisons were made together.

## 3. Results and Discussion

### 3.1. 5G3 Is a Mesenchymal Cell

**Figure 1:**
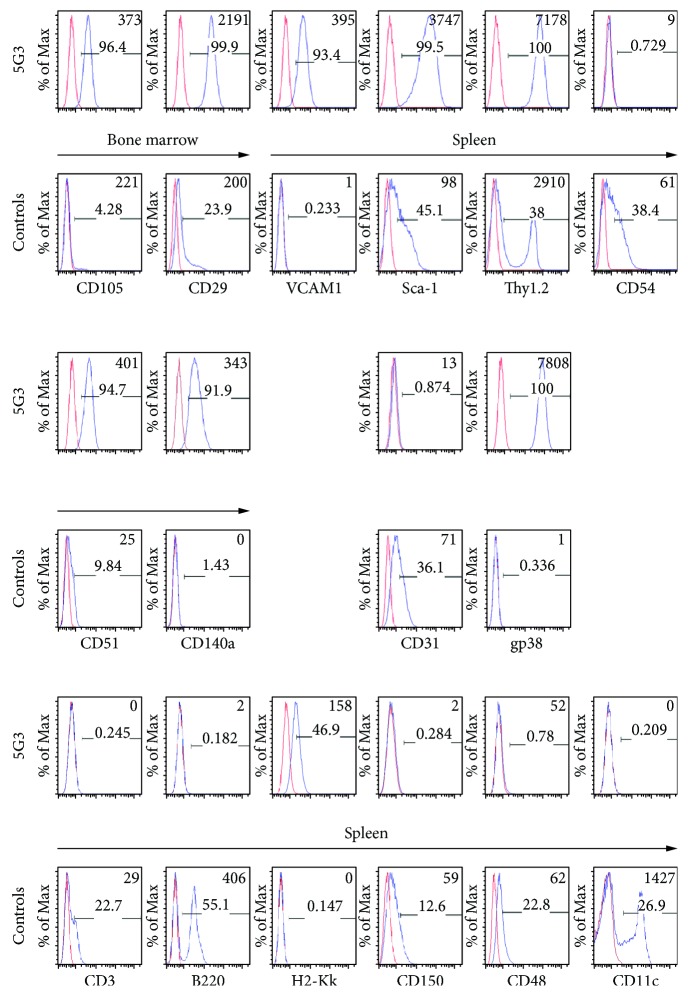
Identification of 5G3 lineage origin. Cells were stained with antibodies to mesenchymal, endothelial, and hematopoietic cell markers. Red histograms show isotype controls, and blue show specific antibody binding. MFI is shown in the top right corner of plots, and % specific staining is shown at the centre. Spleen and bone marrow cells were used as controls.

**Figure 2:**
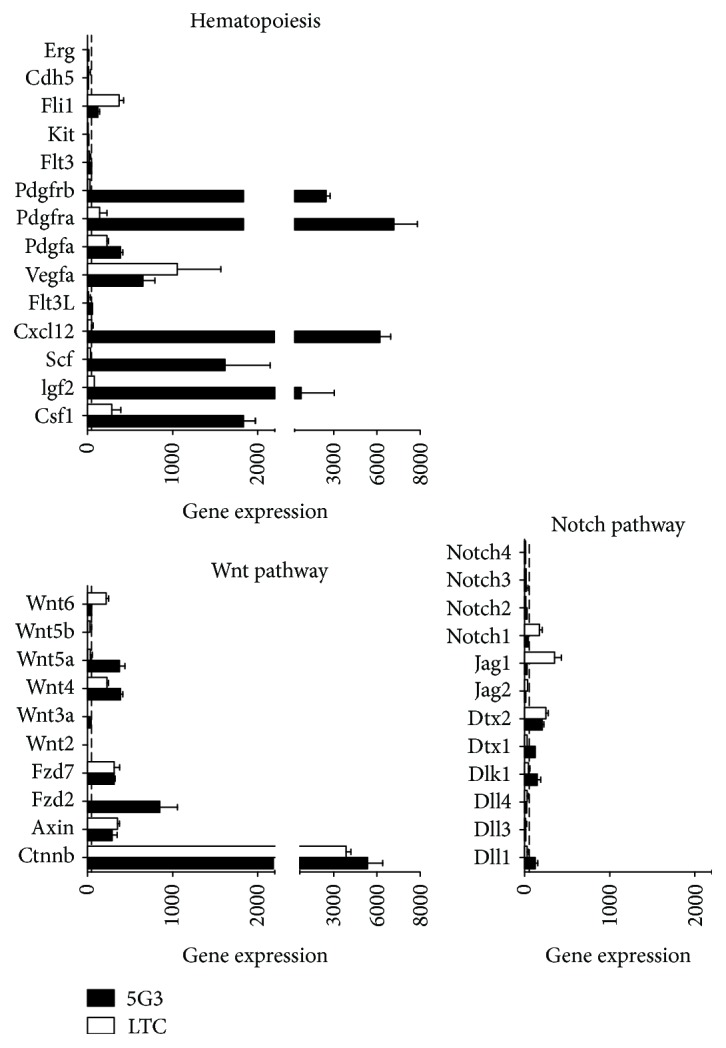
Genes expressed by 5G3. Gene expression was measured by transcriptome analysis using Affymetrix murine genome 430 v.2 genechips and is represented by ln_2_ signal values obtained from two independent experiments (mean ± SE). The cutoff value of 25 is shown as a dashed line. Expression of pathway-specific genes by 5G3 stroma was compared with hematopoietic cells collected from long-term stromal cocultures (LTC).

To characterize the lineage origin of 5G3, cells were stained with antibodies specific for cell surface markers. Stroma was shown to express CD105, CD29, Sca-1, and Thy1 in common with mesenchymal stem cells. Cells also expressed the VCAM1, CD51, and CD140a (PDGFRA) markers of perivascular reticular cells (Figure 1). They are also stained for gp38, a known marker of fibroblastic reticular cells [18]. 5G3 did not express the CD31 or CD54 endothelial markers (Figure 1). That 5G3 stroma is not endothelial and was confirmed by transcriptome analysis in Figure 2 which showed no expression of *Cdh5*, *Fli-1*, or *Erg* [19, 20]. 5G3 also expressed no markers of hematopoietic cells including myeloid and lymphoid subsets (Figure 1).

### 3.2. 5G3 Stroma Supports Myelopoiesis

**Figure 3:**
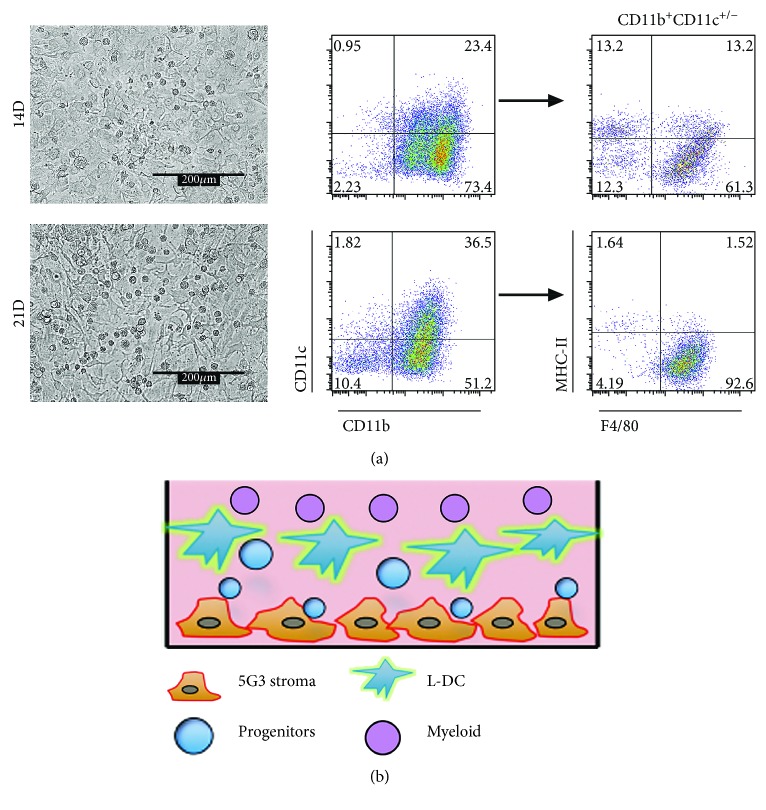
Characterization of cells produced in stromal cocultures of Lin^−^ bone marrow cells over 5G3 stroma. (a) Production of nonadherent cells at 14 and 21 days. Nonadherent cells were collected from the supernatant and stained with antibodies specific for CD11b, CD11c, MHC-II, and F4/80 for flow cytometric analysis to delineate subset type. L-DC was gated as CD11b^+^CD11c^+/−^F4/80^+^MHC-II^−^, cDC-like cells as CD11b^+^CD11c^+/−^F4/80^−^MHC-II^+^, and immature myeloid cells as CD11b^+^CD11c^+/−^F4/80^−^MHC-II^−^. Progenitors were enriched in the CD11b^−^CD11c^−^F4/80^−^MHC-II^−^ subset. One coculture representative of multiple experiments is shown. (b) Diagrammatic representation of cell types characterized in cocultures of Lin^−^ bone marrow over 5G3 stroma.

The hematopoietic support capacity of 5G3 was demonstrated in cocultures grown for up to 7 weeks involving overlay of Lin^−^ bone marrow cells [7] (Figure 3(a)). A history of work from this lab shows that such cocultures produce four main cell types [7, 8, 17, 21, 22]. A diagrammatic representation of cells produced in stromal cocultures overlaid with Lin^−^ bone marrow is shown in Figure 3(b). Multiple experiments over 10 years have identified no production of T, B, or erythroid cells, but long-term production of myeloid dendritic cells is sustained since cocultures maintain myeloid progenitors [7, 8, 16, 17].

Nonadherent cells produced in cocultures were collected from the supernatant and stained with antibodies specific for CD11b, CD11c, MHC-II, and F4/80, for flow cytometric identification of cells produced. The total myeloid population was first gated as a CD11b^+^CD11c^+/−^ population. The L-DC population was then gated as CD11b^+^CD11c^+/−^F4/80^+^MHC-II^−^ cells, conventional (c)DC-like cells as CD11b^+^CD11c^+/−^F4/80^−^MHC-II^+^ cells, and immature myeloid cells or “progenitors” as CD11b^+^CD11c^+^/^−^F4/80^−^MHC-II^−^ (Figure 3(a)). The CD11b^−^CD11c^−^F4/80^−^MHC-II^−^ population is not a pure population of progenitors but is enriched for myeloid progenitors and precursors. After 14 days, L-DC constituted 61.3% of cells produced, with cDC-like cells (13.2%) and myeloid cells (12.3%) as minor populations (Figure 3(a)). After 21 days, a dominant population of L-DC was evident, representing 92.6% of cells, with minor populations of myeloid cells (4.2%) and putative progenitors (10.4%) also evident (Figure 3(a)). cDC-like cells and myeloid cells are produced only transiently from precursors present in Lin^−^ bone marrow and disappear by 21 to 28 days of culture [7, 8, 21].

### 3.3. Wnt Signaling Controls Production of Myeloid Cells in Cocultures

Gene profiling was performed in two replicate experiments comparing 5G3 stroma with cells produced in LTC. For these experiments, 5G3 cells were grown and passaged thrice before RNA collection, and LTC were established for 6 weeks before collection of total cells for RNA preparation. Based on transcriptome analysis, 5G3 stroma expresses Wnt ligands (specifically *Wnt5a*) and Wnt receptors including Frizzled receptor 2 (*Fzd2*) and Frizzled receptor 7 (*Fzd7*) (Figure 2). Control hematopoietic cells expressed *Fzd7* and *Wnt6* (Figure 2). Both 5G3 and hematopoietic cells expressed high levels of *Ctnnb* which encodes *β*-catenin, an essential transcriptional regulator of Wnt signaling, and *Axin* which regulates *β*-catenin. To assess whether Wnt signaling was important for *in vitro* hematopoiesis, two different inhibitors were added into cocultures established with Lin^−^ bone marrow cells. XAV939 disrupts Wnt signaling by degrading *β*-catenin [23]. WntC59 is a potent inhibitor of PORCN required for Wnt palmitoylation, thereby blocking activation of Wnt family proteins [24].

**Figure 4:**
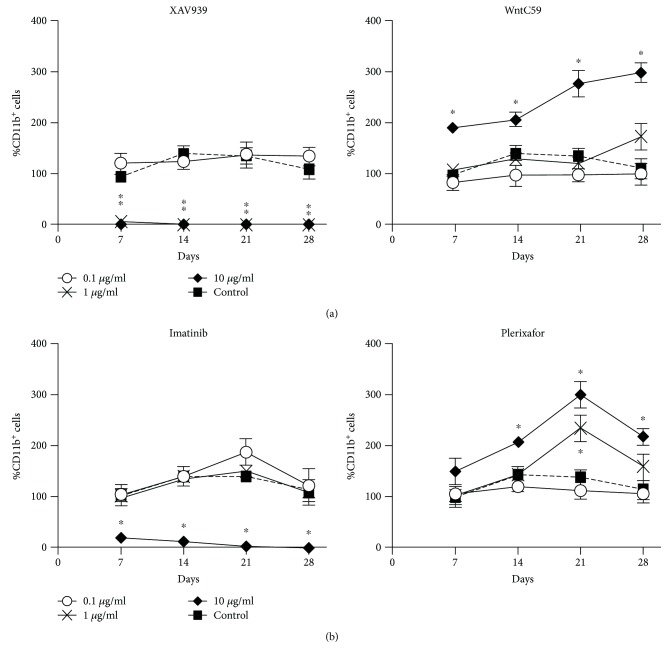
Signaling pathways which control hematopoiesis. Cocultures were established for 28 days by overlay of Lin^−^ bone marrow cells over 5G3 stroma. (a) Soluble inhibitors of *β*-catenin (XAV939) and Wnt activation (WntC59) were added into triplicate flasks. (b) Imatinib and Plerixafor were added into cocultures to assess the effect on cell production over time. In (a) and (b), data shows % myeloid (CD11b^+^) cells produced relatively to input cell number using flow cytometry. Data reflect the mean ± SE (*n* = 3). ∗ annotates inhibitor concentration where cell production is significantly different from controls which contained no inhibitor. Data shown are reflective of two similar experiments.

XAV939 treatment gave a significant decrease in cell production compared with controls across all time points at 10 *μ*g/ml and 1 *μ*g/ml but was ineffective at lower concentrations (Figure 4(a)). XAV939 acts on *β*-catenin, essential for cell survival and proliferation [25]. In contrast, the WntC59 inhibitor gave significantly increased cell production across all time points at a concentration of 10 *μ*g/ml but had no effect at lower concentrations (Figure 4(a)). WntC59 blocks the activation of Wnt family proteins expressed on 5G3 and may release progenitors from a state of maintenance and self-renewal, so giving increased differentiation of myeloid cells. *β*-Catenin and other Wnt proteins are also expressed by hematopoietic cells isolated from cocultures, so that inhibitors may be acting on either or both hematopoietic and stromal cells (Figure 2). However, in the case of Wnt signaling, the specific cell target of inhibition is secondary to evidence that Wnt signaling controls hematopoiesis in this system.

### 3.4. Imatinib Inhibits Myelopoiesis in 5G3 Stromal Cocultures

Gene profiling of 5G3 revealed the expression of genes encoding growth factors like SCF, CXCL12, PDGFA, VEGFA, IGF2, and CSF1 but not FLT3L, CSF2, or CSF3 (Figure 2). The expression of *Cxcl12* and *KitL* (CSF) is consistent with the hypothesis that 5G3 reflects a perivascular reticular cell type in the spleen. To investigate a role for SCF or CXCL12 in hematopoiesis *in vitro*, the specific inhibitors Imatinib and Plerixafor were titrated into cocultures established with Lin^−^ bone marrow cells. Plerixafor is an antagonist of the CXCR4 receptor for CXCL12. However, Plerixafor-treated cocultures gave continuous cell production in all treated cocultures compared with controls across a range of concentrations (Figure 4(b)). No inhibition of cell production was achieved, but enhancement of cell production was achieved over time, giving significantly higher cell production in cultures treated with 10 *μ*g/ml and 1 *μ*g/ml (Figure 4(b)). This increase could relate to the role of CXCL12 in HSC migration, such that inhibition of migration may lead to increased differentiation over time. Imatinib is a selective tyrosine kinase inhibitor of the c-KIT receptor for SCF, and it also inhibits other tyrosine kinases including PDGFR (CD140) and FLT3 [26]. The production of ligands PDGFA and VEGFA by hematopoietic cells (Figure 2) suggests that PDGFRA and PDGFRB could also be targets for Imatinib inhibition. A role for FLT3/FLT3L interaction is not indicated as important based on the absence of gene expression (Figure 2). Addition of Imatinib at 10 *μ*g/ml significantly inhibited cell production from 7 days of culture (Figure 4(b)). Imatinib would appear to act by slowing cell development at the level of progenitors, perhaps inhibiting self-renewal, maintenance, or survival. Imatinib could be inhibiting through binding to c-KIT on progenitors or through binding to PDGFRA or PDGFRB (CD140a/b) expressed by stromal cells. Inhibition of either or both of these signaling pathways is consistent with cell production through hematopoiesis.

### 3.5. Role for Notch Signaling in Hematopoiesis in Cocultures

**Figure 5:**
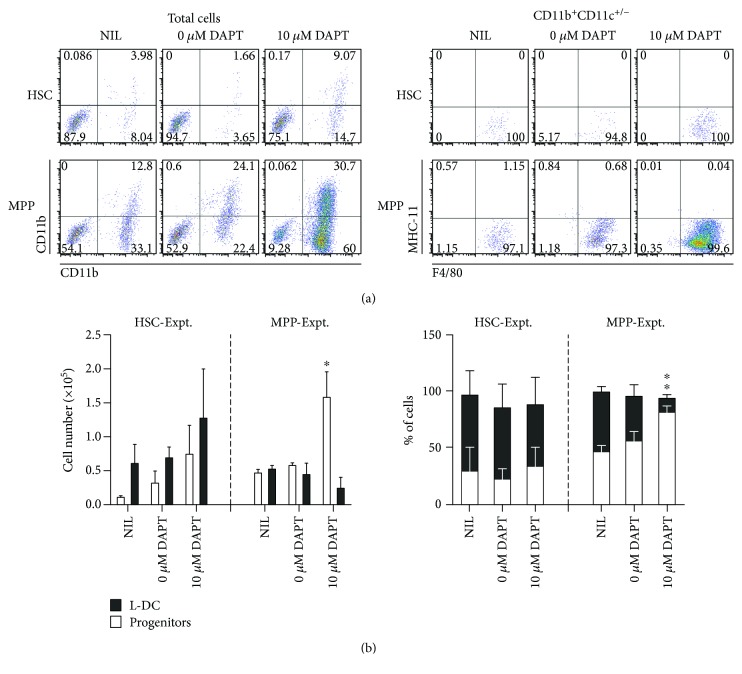
The specific effect of Notch inhibition on hematopoiesis *in vitro*. LT-HSC (Lin^−^Sca-1^+^c-Kit^+^Flt3^−^CD150^+^) and MPP (Lin^−^Sca-1^+^c-Kit^+^Flt3^+^CD150^−^) were sorted from murine bone marrow and cultured above 5G3 stroma for a period of 21 days. Cocultures were supplemented twice weekly with DAPT (10 *μ*M in DMSO), a *γ*-secretase inhibitor. Control cultures received DMSO diluent alone or NIL. Nonadherent cells were collected after 21 days and stained with antibodies specific for CD11b, CD11c, MHC-II, and F4/80 to delineate subsets. (a) Cells were delineated through marker expression as either L-DC (CD11b^+^CD11c^+/−^MHC-II^−^F4/80^+^) or “progenitors” (CD11b^−^CD11c^−^MHC-II^−^F4/80^−^). Staining data of one representative experiment shows the production of predominantly L-DC. (b) The proportional representation of L-DC and the absolute number of L-DC produced for three replicate experiments involving separate cell sorts are shown. Data reflect the mean ± SE (*n* = 3). ∗ identifies a significant difference (*p* ≤ 0.05) in cell production in DAPT treated cocultures (10 *μ*M) compared with NIL controls, except for % of L-DC which is significantly different from both NIL and diluent controls.

Gene profiling in Figure 2 identified several Notch pathway genes expressed by stroma including *Dll1*, *Dlk1*, and *Dtx2* and by hematopoietic cells, namely, *Dtx2*, *Jag1*, and *Notch1.* A role for Notch signaling in *in vitro* hematopoiesis was therefore investigated. The *γ*-secretase indirect Notch inhibitor, DAPT, was used since it abolishes signaling by preventing cleavage of the Notch intracellular domain and translocation into the nucleus for gene activation. In trial experiments of Lin^−^ bone marrow cocultured over 5G3, the number of L-DC produced was found to be noticeably higher in cocultures treated with DAPT at 10.0 *μ*M. Subsets of L-DC, cDC-like cells, and myeloid cells and the CD11b^−^CD11c^−^ subset enriched for progenitors were enumerated. This was consistent with earlier findings which showed that cocultures established with HSC and MPP over 5G3 produce only L-DC and no other myeloid or DC subsets, although they did maintain a progenitor population [17]. The inhibitory effect of 10.0 *μ*M DAPT was therefore tested in cocultures of LT-HSC and MPP over 5G3. L-DC were gated as CD11b^+^CD11c^+/−^F4/80^+^MHC-II^−^ cells and “progenitors” as CD11b^−^CD11c^−^F4/80^−^MHC-II^−^ cells, with no myeloid cells (CD11b^+^CD11c^+/−^MHC-II^−^F4/80^−^) or cDC-like cells (CD11b^+^CD11c^+/−^MHC-II^+^F4/80^−^) produced. The gating strategy used is shown in Figure 5(a).

Significantly higher numbers of L-DC were produced in MPP cocultures given 10.0 *μ*M DAPT compared with controls (Figure 5(b)). It is well known that Notch, along with Wnt signaling, is necessary to maintain HSC and MPP in an undifferentiated state [27]. The observed increase in L-DC production and drop in progenitor numbers in MPP cocultures suggests that Notch signaling may be required for the maintenance of MPP in 5G3 stromal cocultures such that inhibition leads to differentiation and greater production of L-DC. In contrast, DAPT did not affect cell production in HSC cocultures [2, 28]. One explanation could relate to signaling redundancy, such that 5G3 stroma provides an array of signaling molecules for HSC but a more restricted signaling repertoire for MPP. Blocking Notch signaling could have a more profound effect on L-DC production from MPP than from HSC. Previous work from this lab showed that L-DC can derive from both HSC and MPP and that HSC give rise to MPP in cocultures with the development of L-DC being a FLT3-dependent process [17].

## 4. Conclusions

Evidence for specific support for *in vitro* hematopoiesis by spleen stromal lines raises the possibility that *in vivo* spleen contains niches for hematopoietic stem/progenitor cells. The description of the splenic stromal line 5G3 as a perivascular reticular cell now supports that hypothesis in line with similar niches in the bone marrow. 5G3 clearly expresses mesenchymal markers and is closely aligned with CXCL12-abundant reticular cells described in the bone marrow in terms of cell surface markers and gene expression [5]. While the phenotype of cultured cells might differ from their *in vivo* counterparts, cell line models taken in context do provide important information. Here, we now define a specific role for 5G3 stroma in *in vitro* hematopoiesis, with evidence for at least Notch, Wnt, c-KIT/SCF, and PDGFR/VEGF signaling.

## Data Availability

The data used to support the findings of this study are available from the corresponding author upon request.

## Supplementary Materials

Supplementary Materials Supplementary Figure 1: stromal cells do not express hematopoietic cell markers. Supplementary Figure 2: the specific effect of Notch inhibition on hematopoiesis in vitro.

## References

[1] Ding L., Saunders T. L., Enikolopov G., Morrison S. J. (2012). Endothelial and perivascular cells maintain haematopoietic stem cells. *Nature*.

[2] Corselli M., Chin C. J., Parekh C. (2013). Perivascular support of human hematopoietic stem/progenitor cells. *Blood*.

[3] Ogawa M., Matsuzaki Y., Nishikawa S. (1991). Expression and function of c-kit in hemopoietic progenitor cells. *The Journal of Experimental Medicine*.

[4] Leary A. G., Zeng H. Q., Clark S. C., Ogawa M. (1992). Growth factor requirements for survival in G0 and entry into the cell cycle of primitive human hemopoietic progenitors. *Proceedings of the National Academy of Sciences*.

[5] Sugiyama T., Kohara H., Noda M., Nagasawa T. (2006). Maintenance of the hematopoietic stem cell pool by CXCL12-CXCR4 chemokine signaling in bone marrow stromal cell niches. *Immunity*.

[6] Luis T. C., Weerkamp F., Naber B. A. E. (2009). Wnt3a deficiency irreversibly impairs hematopoietic stem cell self-renewal and leads to defects in progenitor cell differentiation. *Blood*.

[7] Periasamy P., Tan J. K. H., Griffiths K. L., O'Neill H. C. (2009). Splenic stromal niches support hematopoiesis of dendritic-like cells from precursors in bone marrow and spleen. *Experimental Hematology*.

[8] Periasamy P., Petvises S., O’Neill H. C. (2013). Development of two distinct dendritic-like APCs in the context of splenic stroma. *Frontiers in Immunology*.

[9] Tan J. K. H., Quah B. J. C., Griffiths K. L., Periasamy P., Hey Y. Y., O’Neill H. C. (2011). Identification of a novel antigen cross-presenting cell type in spleen. *Journal of Cellular and Molecular Medicine*.

[10] Despars G., Ni K., Bouchard A., O'Neill T. J., O'Neill H. C. (2004). Molecular definition of an in vitro niche for dendritic cell development. *Experimental Hematology*.

[11] Despars G., Periasamy P., Tan J., Abbey J., O’Neill T. J., O’Neill H. C. (2008). Gene signature of stromal cells which support dendritic cell development. *Stem Cells and Development*.

[12] Greenwood-Goodwin M., Yang J., Hassanipour M., Larocca D. (2016). A novel lineage restricted, pericyte-like cell line isolated from human embryonic stem cells. *Scientific Reports*.

[13] Ni K., O'Neill H. C. (1997). Long-term stromal cultures produce dendritic-like cells. *British Journal of Haematology*.

[14] O'Neill H. C., Wilson H. L., Quah B., Abbey J. L., Despars G., Ni K. (2004). Dendritic cell development in long-term spleen stromal cultures. *Stem Cells*.

[15] Despars G., O'Neill H. C. (2006). Heterogeneity amongst splenic stromal cell lines which support dendritic cell hematopoiesis. *In Vitro Cellular & Developmental Biology Animal*.

[16] Despars G., O'Neill H. C. (2006). Splenic endothelial cell lines support development of dendritic cells from bone marrow. *Stem Cells*.

[17] Petvises S., O’Neill H. C. (2014). Distinct progenitor origin distinguishes a lineage of dendritic-like cells in spleen. *Frontiers in Immunology*.

[18] Mueller S. N., Germain R. N. (2009). Stromal cell contributions to the homeostasis and functionality of the immune system. *Nature Reviews Immunology*.

[19] Giannotta M., Trani M., Dejana E. (2013). VE-cadherin and endothelial adherens junctions: active guardians of vascular integrity. *Developmental Cell*.

[20] Hewett P. W., Nishi K., Daft E. L., Clifford Murray J. (2001). Selective expression of erg isoforms in human endothelial cells. *The International Journal of Biochemistry & Cell Biology*.

[21] Periasamy P., O'Neill H. C. (2013). Stroma-dependent development of two dendritic-like cell types with distinct antigen presenting capability. *Experimental Hematology*.

[22] Petvises S., O’Neill H. C. (2014). Characterisation of dendritic cells arising from progenitors endogenous to murine spleen. *PLoS One*.

[23] Huang S.-M. A., Mishina Y. M., Liu S. (2009). Tankyrase inhibition stabilizes axin and antagonizes Wnt signalling. *Nature*.

[24] Proffitt K. D., Madan B., Ke Z. (2013). Pharmacological inhibition of the Wnt acyltransferase PORCN prevents growth of WNT-driven mammary cancer. *Cancer Research*.

[25] Ding Y., Su S., Tang W. (2014). Enrichment of the *β*-catenin-TCF complex at the S and G2 phases ensures cell survival and cell cycle progression. *Journal of Cell Science*.

[26] Bartolovic K., Balabanov S., Hartmann U. (2004). Inhibitory effect of imatinib on normal progenitor cells in vitro. *Blood*.

[27] Duncan A. W., Rattis F. M., DiMascio L. N. (2005). Integration of Notch and Wnt signaling in hematopoietic stem cell maintenance. *Nature Immunology*.

[28] Varnum-Finney B., Halasz L. M., Sun M., Gridley T., Radtke F., Bernstein I. D. (2011). Notch2 governs the rate of generation of mouse long- and short-term repopulating stem cells. *The Journal of Clinical Investigation*.

